# Ventricular function and volume assessment in children, adolescents and young adults with thalassemia major without myocardial iron overload

**DOI:** 10.1186/1532-429X-11-S1-P1

**Published:** 2009-01-28

**Authors:** Juliano L Fernandes, Matheus Avelar, Monica Verissimo, Otavio Coelho

**Affiliations:** 1 University of Campinas (Unicamp), Campinas, Brazil; 2 Instituto Boldrini, Campinas, Brazil; 3 Centro Infantil Boldrini, Campinas, Brazil

**Keywords:** Cardiovascular Magnetic Resonance, Body Surface Area, Thalassemia, Chelation Therapy, Thalassemia Major

## Introduction

Reference ranges for normal ventricular function and volumes in patients with thalassemia major without myocardial iron overload have been established before in European countries. However, these values might not be directly applicable to patients in most other countries where they tend to be younger and start chelation therapy later in life.

## Purpose

To study children, adolescents and young adults with thalassemia major with normal T2* values and compare the results to matched normal volunteers as well as patients with high ferritin levels, normal myocardial iron and chronic anemia due to other etiologies.

## Methods

We selected 25 patients (52% male) with thalassemia major (TM) and normal myocardial iron concentrations (T2* > 20 msec) and compared them to 17 age- and gender-matched normal (NL) volunteers (41% male) and 24 gender-matched (58% male) patients with high ferritin levels from other etiologies (NT). All patients underwent a cardiovascular magnetic resonance (CMR) function study using a steady-state free precession sequence. Normalized data to body surface area was compared among the three groups.

## Results

The mean age of TM patients was 18.8 ± 2.3 years (range 7–31) with no significant differences from NL volunteers (16.2 ± 2.8 years, range 5–34, P = NS), but younger than NT patients (39.5 ± 2.4 years, range 11–69, P < 0.001). T2* in patients with TM were somewhat lower than NT patients (27.5 ± 4.3 versus 31.7 ± 6.8, P = 0.06) but still within the normal range. Body surface area was similar in the three study groups (TM, 1.51 ± 0.32 m2; NL, 1.45 ± 0.37 m2; NT, 1.61 ± 0.33 m2; P = NS). Left ventricular ejection fraction was not different when comparing TM and NL patients [65.2 ± 6.0% (95% CI 62.7–67.7), versus 65.4 ± 5.6% (95% CI 62.6–68.3), P = NS], with both values non significantly lower than NT patients [68.5 ± 6.9% (95% CI 65.6–71.4), P = 0.15]. Despite that, normalized diastolic and systolic volumes were higher in patients with TM compared to NL volunteers (78.5 ± 15.8 × 27.4 ± 8.3 ml/m2 versus 63.8 ± 10.4 × 21.8 ± 4.6 ml/m2, P = 0.007 and P = 0.05 respectively) with no significant differences compared to NT patients (75.6 ± 16.3 × 24.8 ± 8.2 ml/m2, P = NS). Normalized mass also showed similar characteristics with higher values in TM patients compared to NL (56.9 ± 10.6 g/m2 versus 37.9 ± 7.9 g/m2, P < 0.001) and no differences compared to NT individuals (62.9 ± 14.2 g/m2, P = NS). Male and female comparisons showed similar results although no differences were found in normalized volumes when looking only in female patients.

## Conclusion

Younger patients with TM do not present different left ventricular function values compared to normal controls despite having increase ventricular volumes and mass. The parameters presented by these patients are similar to older individuals with comparable degrees of chronic anemia. Previously published reference ranges for TM may not be applicable to younger patients with different clinical settings.

